# Australian experience of peptide receptor radionuclide therapy in lung neuroendocrine tumours

**DOI:** 10.18632/oncotarget.27659

**Published:** 2020-07-07

**Authors:** Lisi Elizabeth Lim, David L. Chan, David Thomas, Yang Du, Gary Tincknell, Anna Kuchel, Alexander Davis, Dale L. Bailey, Nick Pavlakis, Gabrielle Cehic, William Macdonald, David Wyld, Eva Segelov

**Affiliations:** ^1^Department of Medical Oncology, Monash Health, Melbourne, Australia; ^2^Department of Medical Oncology, Royal North Shore Hospital, Sydney, Australia; ^3^Bill Walsh Translational Cancer Research Laboratory, Kolling Institute, University of Sydney, Sydney, Australia; ^4^Department of Medical Oncology, St. George Hospital, Sydney, Australia; ^5^Department of Nuclear Medicine, The Queen Elizabeth Hospital, Adelaide, Australia; ^6^Department of Medical Oncology, Royal Brisbane and Women's Hospital, Brisbane, Australia; ^7^Faculty of Medicine, University of Queensland, Brisbane, Australia; ^8^Sydney Vital Translational Cancer Research Centre, Royal North Shore Hospital, Sydney, Australia; ^9^Faculty of Medicine and Health, University of Sydney, Sydney, Australia; ^10^University of South Australia, Adelaide, Australia; ^11^Department of Nuclear Medicine, Fiona Stanley Hospital, Perth, Australia; ^12^Faculty of Medicine, Monash University, Melbourne, Australia

**Keywords:** lung, carcinoid, atypical, neuroendocrine, peptide receptor radionuclide therapy

## Abstract

Background: Peptide receptor radionuclide therapy (PRRT) is an approved treatment modality for gastroenteropancreatic neuroendocrine tumours (GEP NETs), Although Phase III randomised clinical trial data is not available for NETs of other site of origin, in practice, PRRT is used more widely in clinical practice, based on its mechanism of targeting the somatostatin receptor. Use of PRRT for lung (bronchial) NET, specifically typical and atypical carcinoid (TC, AC), has been reported only in small retrospective case series. This multicentre study adds to the evidence regarding utility of PRRT for lung NETs.

Materials and Methods: A retrospective chart review of patients with TC and AC who received ^177^Lu-dotatate PRRT between January 2002 and June 2019 in six hospitals across Australia was undertaken. Data regarding demographics, efficacy and toxicity was evaluated at each site by the treating clinician.

Results: Forty-eight patients (32 AC, 15 TC, 1 unclassified) received a median of four ^177^Lu-dotatate treatments. There was a median of one prior line of systemic treatment (range: 0–3). The response rate to ^177^Lu-dotatate was 33%, with a median overall survival of 49 months (range of 3–91), at a median follow up of 33 months. This compares favourably with GEP NET. Overall toxicity was recorded as modest.

Conclusions: ^177^Lu-dotatate PRRT in patients with lung NETs is used in real world practice, where it appears well-tolerated with some efficacy. Further evidence could be obtained through a global prospective clinical or registry trial.

## INTRODUCTION

Neuroendocrine tumours (NETs) are uncommon malignancies, comprising 0.5% of all cancers [1, 2]. This widely heterogenous group of malignancies arise from neuroendocrine cells that are found in nearly every organ. The gastrointestinal tract is the most common primary site, accounting for around 65% of all NETs, but this includes many small tumours found incidentally on imaging or endoscopy. Lung is the primary site for approximately 20–25% of NETs [3]; conversely NETs comprises about 2% of all lung malignancies [4]. In an Australian series, lung NETs comprised 19% of all NETs (including appendiceal) diagnosed over a 30 year period [5].

Pathological classification of lung (bronchial) NETs continues with the nomenclature of typical carcinoid (TC) and atypical carcinoid (AC), although there is rationale to move to a unified nomenclature shared by NET from all origins [6, 7]. The American Joint Committee on Cancer/World Health Organisation (AJCC/WHO) classification uses the collective term neuroendocrine neoplasms (NENs), with subcategories NET (Grade 1–2) and neuroendocrine carcinoma (Grade 3) [8]. Poorly differentiated and large cell neuroendocrine carcinomas (NECs) are considered to be separate entities and treated differently.

Metastatic and locally advanced lung NETs, whilst rare tumours, cause significant morbidity and mortality [1, 2, 9]. Treatment options are limited both in levels of evidence and access (including funding) and are often extrapolated from therapies tested in patients with gastroenteropancreatic (GEP) NETs. Randomised controlled trial evidence has been sparse until recently, where the number of trials has expanded. New trials have demonstrated that adequate numbers of patients can be recruited through global collaborations, both for protocols specific to lung NETs and those recruiting patients with NETs from a variety of sites.

Systemic therapy is the mainstay of treatment for advanced lung NETs, though there is no univocal treatment strategy [10]. NETs as a whole are less responsive to chemotherapy, with limited efficacy and further evaluation needed for drugs including temozolomide, capecitabine, 5-fluorouracil, doxorubicin, etoposide and platinum agents [10, 11]. Somatostatin analogues (SSA) are commonly used for advanced lung NETs, without specific evidence until very recently, with publication of the LUNA trial [12]. This phase II study randomised patients with lung and thymic NETs to receive long acting pasireotide, everolimus, or a combination of both agents, and showed a 39.0% disease control rate with pasireotide alone (95% confidence interval (CI): 24.2–55.5)), 33.3% for everolimus alone and 58.5% for the combination. Everolimus was also shown to have efficacy among patients with lung NETs enrolled in the phase III RADIANT-4 trial [13, 14].

Peptide receptor radionuclide therapy (PRRT) is a targeted systemic therapy where a radionuclide-somatostatin analogue complex is delivered to NET cells via cell surface somatostatin receptors (SSTR). Various radionuclides have been used, including the beta emitters Yttrium-90 (^90^Y) and Lutetium-177 (^177^Lu), with novel alpha emitters now also under investigation. PRRT is a firmly established treatment modality for advanced GEP NETs following the publication of the landmark NETTER-1 trial [15, 16], where patients with progressive midgut NET were randomised to receive ^177^Lu-dotatate with ongoing octreotide long-acting repeatable (LAR) therapy, or high dose octreotide LAR alone. The primary endpoint of progression free survival (PFS) was strongly in favour of PRRT, with a hazard ratio (HR) of 0.21 (95% CI: 0.14–0.33; *p* < 0.0001). Median overall survival was 27.4 months in the group treated with high-dose octreotide LAR and had not been reached in the PRRT arm at last study update [16]. There was an acceptable toxicity profile and a positive impact on quality of life (QOL) [16, 17].

The significant benefit for PRRT in midgut NETs has provoked debate about whether randomised trials are required to prove its efficacy in NETs of other site of origin. Unlike GEP NETs, which reliably express SSTR, the expression of STTR in lung NETs varies [18, 19], although this was demonstrated with immunohistochemistry, which has variable validity and does not always correlate with SSTR-positron emission tomography findings. There was no identified relationship with tumour aggressiveness or functional status and no difference in SSTR expression has been seen between AC and TC [19].

Several small retrospective case series (*n* = 22–34) of patients with lung NETs have reported potential benefit from PRRT [20–23]. Response rates and outcomes have varied, as expected with heterogenous populations and retrospective, uncontrolled response review. Larger series using various isotopes have also been reported; the largest demonstrated benefit in 114 patients treated with radiopeptides containing ^90^Y, ^177^Lu or a combination in 45, 48, and 21 patients respectively [24].

NET consensus guidelines either omit specific comment on the use of PRRT in lung NETs [25], or state that imaging with SSTR-PET can assist in identifying patients who may benefit from PRRT [26, 27]. Current National Comprehensive Cancer Network (NCCN) guidelines propose that PRRT should be considered for patients with lung NETs following progression on SSA, if SSTR positive on imaging [28, 29]. The Commonwealth Neuroendocrine Tumour Collaboration (CommNETs) and North American Neuroendocrine Tumor Society (NANETS) Consensus for Lung Neuroendocrine Tumors (LNET) also recommend that PRRT may be an option in patients with SSTR positive tumors, based on international expert opinion [30].

For subtypes of rare diseases, such as lung NETs, retrospective case series remain important for documenting treatment benefits and risks. Due to reimbursement restrictions, PRRT is used in only a limited number of centres in Australia, so that collecting data from such centres provides a picture of real world use. We therefore undertook a retrospective review of patients with lung NETs (AC and TC) treated with ^177^Lu-dotatate in Australian centres.

## RESULTS

Forty-eight patients were identified, of which 30 were male. Median age was 68 years (range: 22–81 years). Patient demographics and tumour characteristics are shown in Table 1.

**Table 1:** Patient and tumour characteristics

	Cohort (*n* = 48)
***Age***	
< 70	28
≥ 70	20
***Histopathology***	
Typical carcinoid	15
Atypical carcinoid	32
Unknown^*^	1
***Ki67 %***	
< 3	15
3–20	31
Unknown	2
Sites of disease^**^	
Liver	37
Bone	36
Lymph nodes	29
Lung	13
Subcutaneous	3
Pleura	3
Brain	3
Adrenal	2
Other (breast, gallbladder, ovary, thyroid)	5
***Prior lines of systemic treatment***	***patients***
0	7
1	32
2	7
≥ 3	2
***Chromogranin A (x ULN^)***	***patients***
< 2	15
≥ 2	30
Unknown	3
***Symptomatic***	***patients***
Yes	42
* Secretory symptoms*	30
* Non-secretory symptoms*	33
***No. of PRRT courses***	***patients***
1	42
2	4
3	2
***No. of PRRT doses per course***	***patients***
≤ 2	4
3	2
4	33
5	1
6	5
≥ 7	3

^*^no accessible histopathology(overseas sample). ^**^multiple sites documented where applicable. ^^^ ***xULN*** = multiples of Upper Limit of Normal in local laboratory.

Thirty-two patients received PRRT for progressive disease; nine patients were treated only for symptom control; seven patients received treatment for both progression and symptoms. One patient who had progessive disease received PRRT plus capecitabine chemotherapy as a neoadjuvant approach prior to curative intent surgery. 42 patients were treated with one single course of PRRT, four had 2 and two had 3 courses. Patients received a median of four doses per course of PRRT (range: 1–10), with one patient who received 10 doses as a single drawn out course of PRRT. The median cumulative radiation dose administered per patient, including that from subsequent courses of PRRT, was 31.9GBq (range: 7.6–49.7GBq).

The median time from diagnosis to first PRRT was 15.5 months (range: 1–206 months). Patients received a median of one prior line of systemic treatment (range: 0–3), prior to PRRT. 31 patients were treated with first line SSA, with a median duration of first line SSA of 18 months (range: 1–52 months). Six patients received PRRT as first line systemic treatment.

Twenty-three patients received systemic therapy during PRRT: 11 received concurrent SSA; eight received concurrent capecitabine chemotherapy and four received concurrent combination capecitabine and temozolomide chemotherapy. Regarding SSA therapy, 36 patients received SSA before PRRT while eight received SSA prior to and continued during PRRT. Three patients were recommenced on SSA only during PRRT.

**Figure 1:**
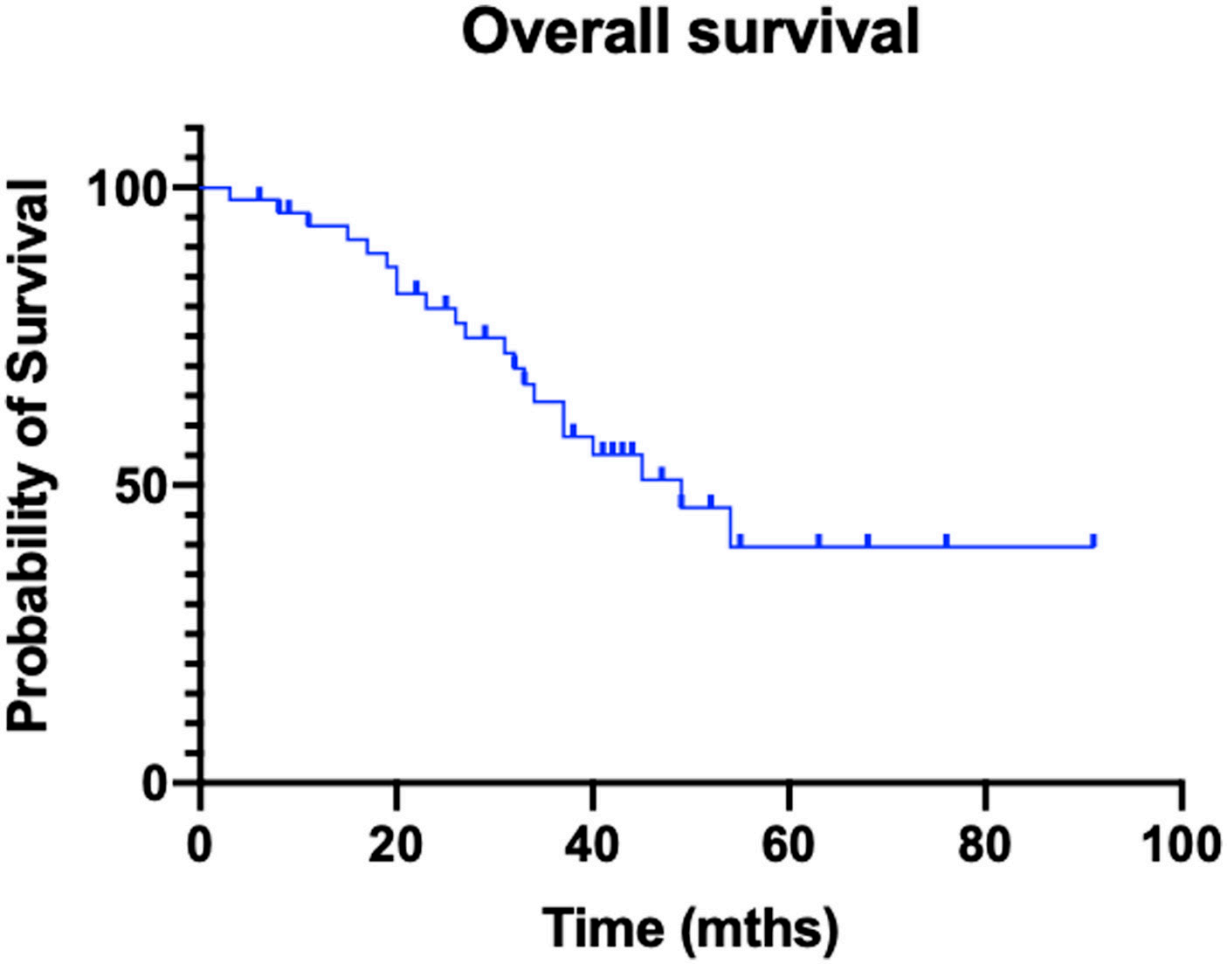
Kaplan–Meier curve of overall survival.

Median follow-up from delivery of the first PRRT dose was 33 months for the entire cohort. Median overall survival was 49 months (OS range: 3–91 months; Figure 1).

Overall response rate was 33% (16 patients), with all responses documented as partial. Stable disease was described in 24 patients, yielding a disease control rate of 83%. Eight patients (17%) were deemed to have progressive disease as best response. Details of the 16 responders are summarised in Table 2.

**Table 2:** Details of responders to PRRT

Patient no.	Months to first systemic treatment post PRRT	Status	Months of follow up from 1st PRRT dose
**1**	-	Alive	43
**2**	-	Deceased	19
**3**	-	Alive	6
**4**	-	Alive	9
**5**	-	Deceased	33
**6**	24	Alive	63
**7**	-	Alive	8
**8**	-	Alive	11
**9**	-	Alive	43
**10**	36	Deceased	45
**11**	-	Alive	41
**12**	36	Alive	49
**13**	36	Alive	91
**14**	-	Alive	52
**15**	-	Alive	76
**16**	8	Alive	68

Two responders were rechallenged with further PRRT as the next line of systemic treatment 36 months post the first course of PRRT, with stable disease as best response. Median overall survival for all responders was 43 months (OS range: 9–91 months; Table 2). Only three of the 13 responders who were alive at data cut-off required further systemic treatment.

With regard to symptoms, post-PPRT there was documented improvement in 14 of 30 patients with secretory symptoms that included flushing and diarrhoea. Fifteen of 33 patients with non-secretory symptoms, including pain and dyspnoea, had documented benefit. No patient experienced an exacerbation of secretory or non-secretory symptoms.

Patients received up to four further lines of systemic treatment after the initial course of PRRT. Twenty-nine patients did not receive any further therapy; respectively, 13, 3, 2 and 1 patient received one, two, three and four further lines; none received more than four. Of the 29 patients who received no further treatment, inclusive of SSA, this was due to ongoing control of disease in the majority (20 patients). Of the six patients who were retreated with subsequent courses of PRRT, two were documented to have a partial response, while the others had stable disease as best response.

### Adverse events

The main toxicities from PRRT included five serious adverse events with two deaths (Table 3).

**Table 3:** PRRT toxicity

Toxicity	Any grade (number of patients)	Grade^*^ 1–2	Grade 3–5
***Haematological***	16	13	2
***Renal***	5	4	1
***Intestinal***	6	5	1
***Hepatic***	2	2	0
***Other*** *(Superior vena cava obstruction)*	1	0	1

^*^CTCAE grading version 4.0; toxicity at any stage during PRRT.

Regarding serious toxicity, one patient who had been heavily pretreated developed acute myeloid leukemia (AML), diagnosed at the time of her first PRRT course, post her second PRRT dose. The AML led to subsequent death. This patient had previously received alkylating chemotherapy (streptozotocin) and 5-fluorouracil, and multiple course of radiotherapy. The development of AML occurred eight to ten weeks post PRRT commencement and approximately four years post streptozotocin.

One death was recorded in a second patient who developed AML. This 81 year old patient, initially developed myelodysplastic syndrome (MDS) 48 months post PRRT, which transformed to lethal AML a year after MDS development. This patient had a normal blood film at PRRT commencement, and received four doses of PRRT with a cumulative dose of 29.35GBq, concurrent with infusional 5-fluorouracil. Prior treatment consisted of SSA only, and cytogenetics were suggestive of therapy related AML.

Acute kidney injury was documented in one patient. This occured during the first PRRT treatment, in the context of carcinoid syndrome with severe diarrhoea resulting in volume depletion. The patient required inpatient admission for intravenous fluids, but recovered fully and proceeded to further PRRT without toxicity. The only serious gastrointestinal adverse event consituted grade 3 nausea which occurred after the first PRRT dose in a single patient who required hospital admission for anti-emetics. There was no recurrence with use of prophylactic anti-emetics in subsequent PRRT doses.

The remaining serious adverse event was the development of superior vena caval obstruction from a thrombus that developed during a course of PRRT. The treatment could not be retrospectively excluded as a contributing factor.

## DISCUSSION

This study represents the largest case series of patients with TC and AC treated with ^177^Lu-dotatate thus contributing to the real world evidence base. For patients with uncommon cancers, especially where treatment options are limited, documenting treatment benefit and toxicity is an important contribution to the knowledge base. Previous reports of the use of PRRT in lung NETs consist of retrospective case series, summarised in Table 4.

**Table 4:** Prior studies of PRRT including patients with lung or bronchial NET^*^

Study	*n* (LNETs)	*n* (other NETs)	Isotope	Median OS	Median PFS	Median TTP	ORR
**Mariniello A, 2016 [24]**	114	-	^90^Y	58.8 m	28.0 m	-	Highest with ^77^Lu at 38.1%
			v ^177^Lu				
			v				
			^90^Y+ ^177^Lu				
**Brabander T, 2017 [31]**	23	420	^177^Lu	63 m	29 m	-	39%
**Horsch D, 2016 [32] (German registry)**	18	396	^90^Y	59 m	41 m	-	28%
			v ^177^Lu				
			v				
			^90^Y+ ^177^Lu				
**Parghane RV, 2017 [21]**	22	-	^177^Lu	40 m		-	63% (3 scale criteria)
**Demirci E, 2019 [33]**	29	157	^177^Lu	-	36.4 m	-	50%
**van Essen M, 2007 [34]**	9	7	^177^Lu	-	-	31 m	50%
**Sabet, 2017 [22]**	22	-	^177^Lu	42 m	27 m	-	27.3%
**Imhof, 2011 [35]**	84	1109	^90^Y	-	-	-	29.7%
**Villard, 2012 [36]**	N/E	486	^90^Y	47.5 v	-	-	-
			v	66.1 m			
			^90^Y+ ^177^Lu				
**Gabriel, 2019 [36]**	4	34	^90^Y	79 m	-	-	-
**Garske-Roman, 2018 [37]**	6	200	^177^Lu	43 m	27 m	-	24%
**Baum, 2018 [38]**	75	1048	^90^Y	40 m (LNETs)	11 m (LNETs)	-	-
			v ^177^Lu				
			v				
			^90^Y+ ^177^Lu				
**Sharma, 2017 [39]**	18	135	^90^Y	-	-	18.6 m (LNETs)	-
			^177^Lu				
**Koffas, 2016 [40] (abstract)**	22	-	^90^Y	26 m	-	14.1 m	-
			^177^Lu				

^*^Studies with ≤ 3 patients with LNET or Grade 3 neuroendocrine carcinomas were excluded. Abbreviations: LNET = Lung neuroendocrine tumour, NET = Neuroendocrine Tumour, N/E = not evaluable, ORR = overall response rate, OS = overall survival, TTP = time to progression.

PRRT is only available at limited centres in Australia, due to Government health reimbursement arrangements. Hence the experience reported here, in six centres across most of the states of Australia, is a reasonable representation of patients with lung NET in the community.

Response rates and median overall survival in our cohort are consistent with the largest study of PRRT in lung NET, where 114 patients with bronchial NET were treated with various isotopes (^90^Y, ^177^Lu, ^90^Y+ ^177^Lu) [24]. Mariniello et al. reported for this series a median overall survival of 58.8 months and objective response rates of 26.5%. Toxicity was also comparable, with good tolerance for PRRT, however, notably the restrospective Mariniello series had no cases of myelodysplasia or leukemia, at comparable median follow up of 45.1 months (range 2–191 months). In addition, the most significant renal toxicity was grade 2 and occurred more commonly with ^90^Y.

Results from a large single centre study using data from the Netherlands Cancer Registry revealed a 4% incidence of persistant haematologic dysfunction, and 2.9% incidence of hematopoietic neoplasms, among 274 patients with GEP NETs who had received ^177^Lu-dotatate [41]. A large single institution restrospective study of 521 patients with NET of various primary sites (1 lung NET) who had received ^177^Lu-dotatate found that therapy-related myeloid neoplasms were an infrequent but serious adverse effect, with an incidence of 4.8% [42], and poor overall survival. Six and nine patients developed AML and MDS respectively. The haematologic events in our 48 patient cohort is in keeping with the incidence seen in this large study and from the Dutch retrospective study.

The limitations of our study are clear. The case numbers are small as with any rare cancer, and patients were treated at multiple sites and over a time period of nearly 20 years. During this time, the histological classification of lung NET changed [43]. However, all patients had a Ki67 of less than 20%, therefore this remains a report of patients with low to intermediate grade NET. Using the patient’s treating physician at each site to collect the data was aimed at providing the most accurate assessment given the retrospective nature of the study. Only overall survival was reported, as date of last follow up and death could be ascertained reliably, and progression free survival was omitted as it could not be reported with the appropriate degree of certainty and would not be interpretable. Timing and schedule of response evaluation varied between patients and between sites, and were not uniformly measured with standardised criteria. Similarly, symptom evaluation was based on retrospective review of medical records by the treating physician at each site and no QOL questionnaires were used. Uniform adoption of QOL assessment would be an ideal component of a prospective trial.

The argument remains as to the need and viability of a dedicated prospective study of PRRT in lung NETs. Ongoing PRRT studies with ^177^Lu, that are agnostic of NET primary site and thus include lung NETs, are the phase II LUTHREE trial (NCT03454763 [44]), exploring two different dosing schedules, and the phase II P-PRRT trial (NCT02754297 [45]), which investigates personalised PRRT. The combination of ^177^Lu-dotatate PRRT and everolimus is also being evaluated in lung and GEP-NETs, in a phase I–II trial (NCT03629847 [46]). Global efforts could ensure maximum participation so that enrolment is sufficient to make useful conclusions. Other radionuclides under evaluation include ²¹²Pb-DOTAMTATE (AlphaMedix™) in SSTR positive NETs of all primary sites in a phase I trial (NCT03466216 [47]).

Further data will be forthcoming also from studies of PRRT in patients with SSTR-expressing tumours of histologies other than NET. The randomised phase II LUTHREE trial [44] is inclusive of all SSTR positive tumour types, and is not restricted to NETs. The POLNETS trial [48] is also extending the use of PRRT to paraganglioma and pheochromocytoma, in addition to advanced NETs of any site of origin.

## MATERIALS AND METHODS

All major Australian NET treatment centres were invited to participate. After ethics and institutional governance approval, patients with TC and AC who received at least 1 dose of ^177^Lu-dotatate between 1 January 2002 and 30 June 2019 were identified from clinical records. Patients with grade 3 tumours or large cell neuroendocrine carcinoma were excluded, as were radionuclides other than ^177^Lu-dotatate. All patients received a renoprotective amino acid infusion prior to intravenous administration of the radiopharmaceutical, using a standard single day protocol [49]. Data was obtained from review of medical records by the treating physician or team member at each site: Monash Health (Victoria), St George and Royal North Shore Hospitals (New South Wales), Royal Brisbane and Women’s Hospital (Queensland), The Queen Elizabeth Hospital (South Australia) and Fiona Stanley Hospital (Western Australia). De-identified data was compiled centrally and queries were referred back to the local site.

### Definitions

Histology was coded as assigned at time of diagnosis. Where classification as either AC or TC was not documented, histopathology was reviewed at the relevant site. Clinical records at or closest to the time of first PRRT were used to source: presence of symptoms, sites of disease (from structural and functional imaging) and a single chromogranin A value. The rationale for treatment was categorised at that time point into: progressive disease, symptom control, or both. Each administration of PRRT was counted as one treatment dose. The first course of PRRT generally comprised two to four sequential treatment doses. Subsequent courses of PRRT were recorded as another line of systemic therapy.

Outcomes related to the initial PRRT course were measured. Overall survival (OS) was measured from the date of delivery of first PRRT until death from any cause. Disease response was based on review by the patient’s treating physician of all contemporaneous notes, radiology reports and correspondence, as the retrospective nature of the study did not allow for control and verification of imaging-based outcome measures. Symptom benefit was also based on review of medical records and QOL questionnaires were not used. Toxicity was graded by Common Terminology Criteria for Adverse Events (CTCAE) criteria (version 4.0) [50]. Adverse events included toxicity were summed from all PRRT administrations.

## CONCLUSIONS

This retrospective study contributes to the growing body of evidence evaluating PRRT in lung NET. In the real world, ^177^Lu-dotatate appears to be a relatively safe and effective option for patients with this rare cancer. It is hoped that the many remaining questions are answered by randomised clinical trials or studies using novel designs such as registry trials in the future.

## Abbreviations

^177^Lu: ^177^Lutetium
90Y: ^90^Yttrium
AC: atypical carcinoid
AJCC: American Joint Committee on Cancer
AML: acute myeloid leukemia
CTCAE: Common Terminology Criteria for Adverse Events
DLT: dose limiting toxicity
GEP: gastroenteropancreatic
LNET: lung neuroendocrine tumour
MDS: myelodysplastic syndrome
MTD: maximum tolerated dose
NCCN: National Comprehensive Cancer Network
NEC: neuroendocrine carcinoma
NEN: neuroendocrine neoplasm
NET: neuroendocrine tumour
N/E: not evaluable
ORR: overall response rate
PD: progressive disease
PFS: progression free survival
PR: partial response
PRRT: peptide receptor radionuclide therapy
QOL: quality of life
SD: stable disease
TC: typical carcinoid
TTP: time to progression
ULN: upper limit of normal
WHO: World Health Organisation
